# Reallocating Time from Sedentary Behavior to Light and Moderate-to-Vigorous Physical Activity: What Has a Stronger Association with Adiposity in Older Adult Women?

**DOI:** 10.3390/ijerph15071444

**Published:** 2018-07-09

**Authors:** Jana Pelclová, Nikola Štefelová, Jana Hodonská, Jan Dygrýn, Aleš Gába, Izabela Zając-Gawlak

**Affiliations:** 1 Faculty of Physical Culture, Palacký University Olomouc, Olomouc 771 11, Czech Republic; jana.hodonska@upol.cz (J.H.); jan.dygryn@upol.cz (J.D.); ales.gaba@upol.cz (A.G.); 2 Faculty of Science, Palacký University Olomouc, Olomouc 771 11, Czech Republic; Nikola.Stefelova@seznam.cz; 3 The Jerzy Kukuczka Academy of Physical Education, 40-065 Katowice, Poland; izazaja@wp.pl

**Keywords:** adiposity, BMI, fat mass, compositional data analysis, movement behaviors, Central Europe

## Abstract

This study is the first to use compositional data analysis to investigate movement behaviors of elderly women and their relationships with fat mass percentage (FM%). The focus of the study is on the associations of time reallocations from sedentary behavior (SB) to light physical activity (LIPA) or moderate-to-vigorous physical activity (MVPA) with adiposity. Over 400 older adult women were recruited as part of the cross-sectionally conducted measurements of older adults aged 60+ in Central European countries. An accelerometer was used to assess daily movement behaviors. Body mass index (BMI) and fat mass percentage (FM%) were assessed as adiposity indicators using InBody 720 MFBIA. Using LS-regression, we found positive relationships of BMI and FM% with SB (relative to remaining movement behaviors) (*p* < 0.001 for both), while their relationship with MVPA (relative to remaining movement behaviors) were negative (*p* < 0.001 for both). The estimated BMI and FM% associated with a 30-min SB-to-MVPA reallocation were reduced by 1.5 kg/m^2^ and 2.2 percentage points, respectively, whereas they were not reduced significantly with the reallocation of 30 min from SB to LIPA. The findings highlight that SB and MVPA, but not LIPA, are significantly associated with adiposity in elderly women. The reallocation of time from SB to MVPA could be advocated in weight loss interventions in older women.

## 1. Introduction

The world’s population is aging [1]. Moreover, the proportion of older adults who are overweight or obese is increasing [2]. These issues, in the context of a lack of or decline in health-enhancing physical activity (PA) and the concurrent rise of sedentary time in the senior population [3], have resulted in an urgent need for health-movement behavior-related studies., especially for those studies that would enable effective solutions to mitigate these negative trends to be found [4,5].

Taking into account daily behaviors such as sleep, sedentary behavior (SB), and activities of different intensities as dependent and time-bounded gives us a new opportunity to examine the combined effect of such behaviors on different health indicators, including the obesity-related ones [6]. This approach acknowledging the compositional nature of time-use data has been recently advocated [7,8] and used in a range of studies that examined cardio-metabolic health [9,10], cardio-respiratory fitness [10], and obesity [10,11,12,13] in various populations.

In previous studies in older adults which did not adequately acknowledged the compositional nature of daily behaviors but instead considered them independent variables, SB has been positively associated with adiposity measures, such as body mass index (BMI), waist-to-hip ratio (WHR), or waist circumference [13], and conversely, moderate-to-vigorous PA (MVPA) has been suggested for the prevention and treatment of adiposity [14,15].

A benefit of using the compositional approach to the analysis of movement behavior data is that it allows to estimate changes in adiposity associated with reallocation of time from one behavior to another or between multiple behaviors. Hence, the key role of reallocation of MVPA being replaced mainly by SB was highlighted in relation to lower adiposity in children [11,12,16,17], adults [9], and older adults [10].

To date, only one study has investigated the relationship between older adults’ reallocation of time between movement/non-movement related time-use components and anthropometric obesity indices such as BMI and WHR [10]. The current study is the first to use compositional data analysis to investigate the movement behavior data of elderly women and its relationship with fat mass percentage (FM%). Mainly, we focus on the associations of time reallocation from SB to light physical activity (LIPA) or MVPA with adiposity. We hypothesized that relatively higher time in MVPA and LIPA and relatively lower SB would be favourably related to obesity in older adults. The aims and design of the current study are in line with the framework for Viable Integrative Research in Time-Use Epidemiology (VIRTUE) [8].

## 2. Materials and Methods 

### 2.1. Study Sample

Over 400 older adult women were recruited as part of the cross-sectionally conducted measurements of older adults in cities in three Central European countries (the Czech Republic, Poland, and Slovakia) between 2008 and 2018. Seniors aged ≥60 years from local senior clubs and programmes of Universities of the Third Age were invited to participate voluntarily in the measurements. The data collection consisted of PA and SB monitoring and body composition analysis.

The exclusion criteria included mobility limitations (major hip or knee surgery in the previous 12 months, amputation, paralysis, etc.) that might have interfered with body composition and PA and SB measurement. Hence, the current study sample consists of 314 older women. All the participants gave their informed consent in a written form before taking part in the study. The study was performed in accordance with the Helsinki Declaration and was approved by the ethics committee of the Faculty of Physical Culture of Palacký University Olomouc (No. 20/2016). 

### 2.2. Physical Activity Assessment

The PA level was assessed as counts/day, which were determined by a hip-mounted GT1M accelerometer (Manufacturing Technology Inc., Fort Walton Beach, FL, USA). Participants were instructed to wear the device for eight consecutive days during waking hours, but not during bathing or swimming activities. Instructions about the use of the accelerometer were given to the participants immediately after they received the device. The accelerometers recorded activity counts in one-minute epochs. Non-wear time was considered as a period of 60 consecutive minutes that detected no movement (0 cpm), allowing for two min of interruptions >0 cmp [18]. This algorithm is provided in the ActiLife software (ActiGraph LLC., Pensacola, FL, USA). Ten hours and ≥4 days of accelerometer wear were considered to be valid data [19]. For each valid day of monitoring, information about SB, LIPA, and MVPA was derived. Each minute of wearing time with <100 cpm was considered SB. On the basis of previously conducted studies [20,21,22,23], Central European women are considered active; therefore, the PA level was defined according to the Freedson cut-off point for the adult population. LIPA was defined as all the minutes in the range 100–1951 cpm, and MVPA as all the minutes with values ≥1952 cpm [24].

### 2.3. Body Adiposity Assessment 

Height was measured to the nearest 0.1 cm using a P-375 portable anthropometer (Trystom, Olomouc, Czech Republic) while the participants were barefooted. Weight was measured to the nearest 0.1 kg. BMI was calculated by dividing body weight (in kilograms) by height (in metres squared). Overweight and obesity were defined as a BMI of ≥25 and ≥30 kg/m^2^, respectively. As BMI might have a lower sensitivity to detect adiposity than fat mass in women [25], FM% was assessed as an adiposity indicator using the InBody 720 multi-frequency bioelectrical impedance analyzer (Biospace Co., Ltd., Seoul, Korea). Multi-frequency bioelectrical impedance is a valid method for the assessment of body composition in both normal-weight and obese elderly women [26]. Cut-off point of 35% for two FM% subgroups (normal vs. excess FM%) was defined.

### 2.4. Assessment of Covariates

Participants completed a basic demographic questionnaire, which gathered information on their age (date of birth), years of education, smoking status and self-rated health (categories of excellent, very good, good, fair, or poor). The variable of self-rated health was dichotomized with first three answers considered as “good health status”.

### 2.5. Statistical Analysis

Time-use data is usually reported in the form of the amount of time spent in different activities (sleep, SB, and PA of various intensities) during some time period. Thus, they represent parts of a whole and meets the properties of compositional data (compositions), i.e., multivariate observations consisting of positive parts carrying relevant information. The compositional parts are co-dependent, which means that change in one or several components necessarily affects the remaining ones. Compositional analysis takes into account the relative scale of the data by focusing on the log-ratios between the parts. Therefore, the results do not depend on whether the data are expressed in proportions, percentages, or in the original units. The log-ratio approach also ensures that it does not matter if only a subset of components (subcompositions) is available for the analysis instead of the whole composition (as in our study, where sleep was not measured). As compositional data can be (not necessarily) represented without loss of information as vectors with an arbitrary chosen constant sum constraint (i.e., closed to some constant) [27], here we can close the data to 16 h, standing for a representation of an ideal active portion of the day.

Three parts of the composition of the waking movement behavior were differentiated in this study—SB, LIPA, and MVPA. First, a compositional variation matrix was computed to obtain the proportional relationships between the parts. Then, compositional means (centres) were calculated for the whole dataset, for the three BMI subgroups and two FM% subgroups. The relative differences between the subgroups were visualized with the use of a compositional mean barplot. Finally, compositional regression of the real response (BMI and FM%, respectively) on the composition of movement behavior was conducted. We controlled the analysis for several factors that have been identified as strong predictors of adiposity. This includes self-reported age [28], education level [29,30], self-rated health [31] and smoking [32]. BMI and FM% were predicted for time reallocation from SB to LIPA and SB to MVPA respectively. Statistical analysis was undertaken using the SPSS 22.0 software (SPSS, Chicago, IL, USA) and R 3.4.2 software (R Foundation for Statistical Computing, Vienna, Austria).

## 3. Results

The characteristics of the study samples are shown in Table 1. According to BMI, 65% of the women were overweight or obese. Over 57% of the women were classified as obese, having > 35% of FM. The average age of the participants was 66.6 years, and no differences in age were found among the women in different BMI and FM% categories (*p* ≥ 0.25 for both comparisons).

**Figure 1:**
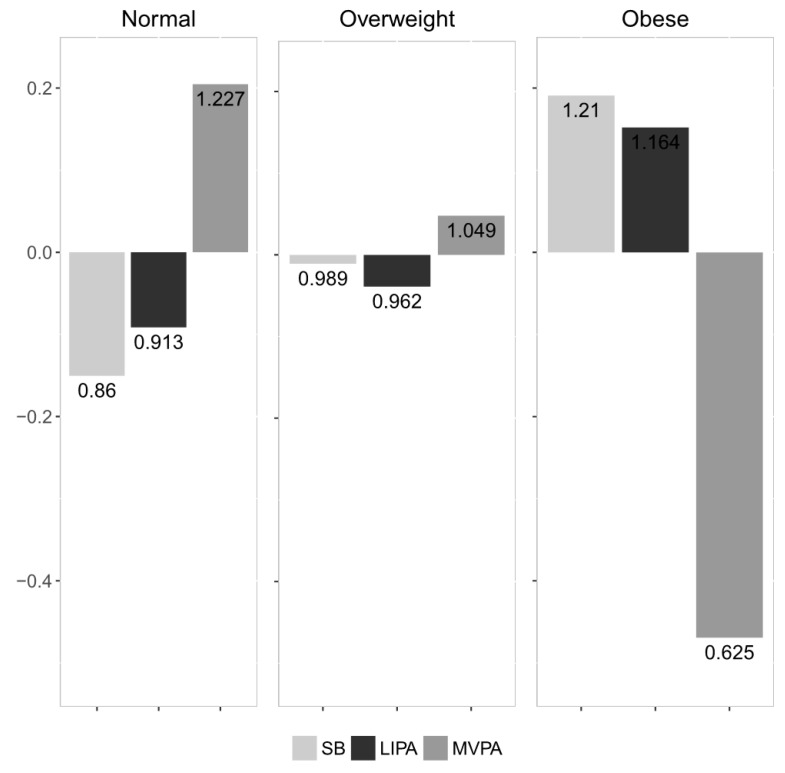
Compositional mean barplot for the BMI categories. The values on the left vertical axis correspond to the log-ratios between the subgroup’s centre and the overall centre after centering the data, whereas the numeric labels of the bars display the actual proportion relative to the overall mean composition.

**Figure 2:**
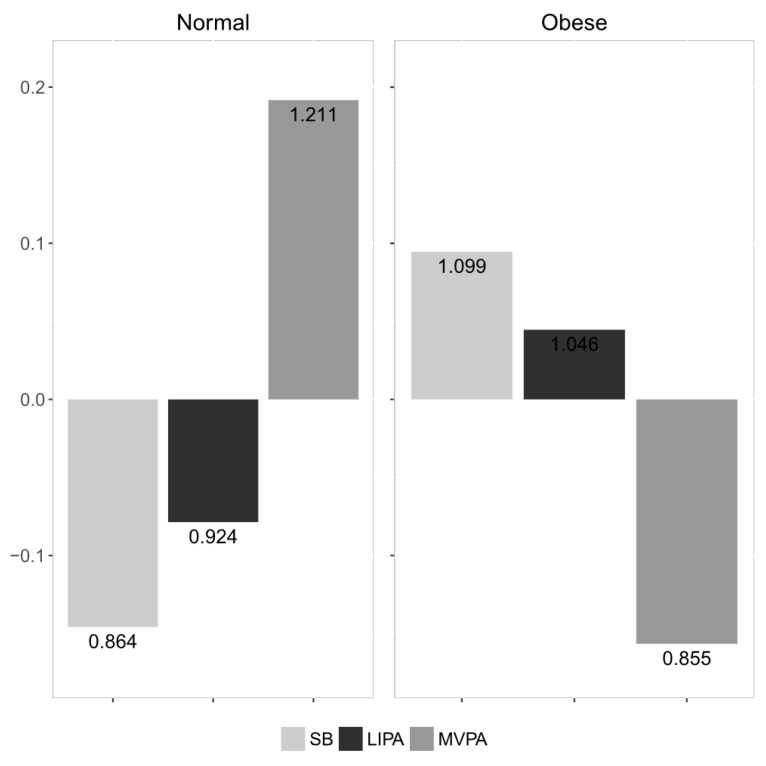
Compositional mean barplots for fat mass percentage (FM%) categories. The values on the left vertical axis correspond to the log-ratios between the subgroup’s centre and the overall centre after centering the data, whereas the numeric labels of the bars display the actual proportion relative to the overall mean composition.

The proportionality between the compositional parts that were considered are summarized in Table 2. The values represent the variance of the respective pair-wise log-ratios. The most proportional parts were SB and LIPA (the lowest value), while the least proportional were SB and MVPA (the highest value). The compositional mean barplot (Figure 1) visualizes the relative differences between the BMI categories. In the normal weight group, the proportion of time spent in MVPA was higher by 22.7% relative to the overall centre, while in the obese group it was reduced by 37.5%. Moreover, the proportion of SB and LIPA was higher by 21% and 16.4%, respectively, in the obese group. Figure 2 shows a compositional mean barplot for the subgroups according to FM%. Here, the proportion of time spent in MVPA in the normal weight group was 21.1% higher relative to the overall centre, while it was reduced by 14.5% in the obese group.

The relationship between BMI (FM%) and movement behavior composition was further examined via compositional LS regression by setting BMI (FM%) as the outcome variable and the composition expressed in pivot coordinates plus age and various factors as explanatory variables. Three models were considered, and the summarized results are displayed in Table 3, where *β* stands for the unstandardized coefficient corresponding to the first pivot coordinate with the respective part in the numerator. The relationships of BMI and FM% with SB (relative to the remaining parts) were positive (*p* < 0.001 for both), while their relationships with MVPA (relative to the remaining parts) were negative (*p* < 0.001 for both).

Table 4 and Table 5 show the estimated BMI and FM% for reallocations of 0 to 30 min from SB to MVPA and LIPA, respectively, starting from the mean composition closed to 16 h. The estimated BMI and FM% associated with a 30-min SB-to-MVPA reallocation are reduced by 1.51 kg/m^2^ and 2.18 percentage points, respectively, whereas they are not reduced significantly even if we reallocate 30 min from SB to LIPA.

## 4. Discussion

This study contributes to the limited body of literature in time-use epidemiology on the relationships between movement behaviors and adiposity in older adults. In a sample of elderly Central European women, a significant relationship between the composition of daily activity and both BMI and FM% was found. Our findings that MVPA is an important activity type within the composition negatively associated with fatness are in line with previous studies across populations, including children and young people [12,16,17], adults [9], and older adults [10]. 

Moreover, we mainly focused on the impact of time reallocation from SB to LIPA or MVPA on adiposity. This aim intentionally touches current public health guidelines recommending at least 150 min of MVPA possibly divided into 30 min of MVPA on most days of the week and minimizing the amount of time spent in prolonged sitting and breaking up long periods of sitting as often as possible [33,34]. Especially in older adults, as the most sedentary population, breaking up sitting time with bouts of LIPA may be readily incorporated into a daily domestic-related lifestyle [35]. Although the potential effect of LIPA on health is not widely documented, there is some evidence that LIPA might be associated with specific health variables [36], including adiposity [37]. Some studies even suggest that the effect of LIPA on some health-related outcomes might be as high as for MVPA of the same duration [38]. In our study, the estimated BMI and FM% associated with a 30-min SB-to-MVPA reallocation were reduced by 1.51 kg/m^2^ and 2.18 %, respectively, whereas they were not reduced significantly even if we reallocate 30 min from SB to LIPA. This is in accordance with a study of older Australian adults [10], where the reallocation of time from different activities to MVPA was consistently moderately to strongly associated with favourable anthropometric obesity indices (BMI and WHR) and in contrast, the reallocation of time from SB to LIPA showed no significant associations. Moreover, estimated BMI associated with 15-min SB-to-MVPA and 15-min SB-to-LIPA reallocations was reduced by 0.7 kg/m^2^ and 0.1 kg/m^2^, respectively, in older Australian adults and by 0.86 kg/m^2^ and 0.07 kg/m^2^, respectively, in elderly Central European women. Hence, similarly to the Australian study of older adults, our study did not support the evidence related to the above-mentioned public health recommendations that the adiposity reduction might appear with replacing SB by LIPA in older adults.

To our knowledge, this is the first study to examine associations between the movement behaviors of elderly women and objectively measured adiposity. A previous study on older adults suggested that the associations of adiposity and movement behaviors were stronger when PA was assessed by an accelerometer compared with a questionnaire [39]. However, a limitation of our study was that using hip-worn accelerometers, we were not able to detect and recognize sitting and quiet standing. Moreover, the accelerometer cut-off points for adults were used as in the study of older Australian adults [10] and to enable the comparison of the results. Although the regression models were adjusted for several confounders, information about chronic respiratory diseases and other comorbidities, as well as dietary information are missing in this study. It should be noted that the duration of sleep was not observed in this study and thus, the composition of waking behaviors closed to 16 h was used. Although this approach is methodologically sound [27], including sleep as the fourth behavior would fit a 24-h composition and would result in additional findings relevant to public health. Furthermore, this study mainly focused on the estimation of adiposity for the reallocation of time from SB to LIPA or MVPA following the current SB guidelines. However, previous studies [9,10,16] have suggested that the effect of MVPA being displaced by SB on adiposity might be even stronger. This remains to be confirmed in future studies among older adults.

## 5. Conclusions

The results of this study contribute to the emerging field of time-use epidemiology. The findings of our compositional data analysis highlight that SB and MVPA, but not LIPA, are behaviors within the composition significantly associated with adiposity in elderly women. The reallocation of time from SB to MVPA could be advocated in weight loss interventions in older women.

## Figures and Tables

**Table 1:** Characteristics of the study sample.

	Mean	SD
*Age and other covariates*		
Age (years)	66.6	6.5
Education (years)	13.1	4.8
Good health status (*n* (% of *n*))	238 (76)	
Smokers (*n* (% of *n*))	14 (4.5)	
*Anthropometrics*		
Body height (cm)	160.9	6.5
Body weight (kg)	70.2	12.0
Body mass index (kg/m^2^)	27.1	4.4
Fat mass percentage (%)	36.1	7.1
*Activity composition* ^a^		
Compositional mean of (SB, LPA, MVPA) in min/16 h	548.54, 376.38, 35.08	
Compositional mean of (SB, LPA, MVPA) in %	57.14, 39.21, 3.65	
*Weight status according to BMI, n (% of n)*		
Normal weight (<25 kg/m^2^)	110 (35.0)	
Overweight (25–29.9 kg/m^2^)	133 (42.4)	
Obesity (≥30 kg/m^2^)	71 (22.6)	
*Obesity status according to FM%, n (% of n)*		
Normal (<35%)	134 (42.7)	
Obesity (>35%)	180 (57.3)	

Note: *n*, number of participants; *SD*, standard deviation; *BMI*, body mass index; *FM%*, fat mass percentage; ^a^ normalized to 16 h.

**Table 2:** Compositional variation matrix of movement behavior composition.

	SB	LIPA	MVPA
SB	0.00	0.19	0.59
LIPA	0.19	0.00	0.51
MVPA	0.59	0.51	0.00

Note: SB, sedentary behavior; LIPA, light physical activity; MVPA, moderate-to-vigorous physical activity.

**Table 3:** LS regression estimates for adjusted compositional models.

	BMI			FM%		
	*β*	*SE*	*p*	*β*	*SE*	*p*
SB	2.80	0.69	<0.001	5.44	1.12	<0.001
LIPA	−0.06	0.72	0.93	−1.61	1.17	0.169
MVPA	−2.74	0.33	<0.001	−3.83	0.53	<0.001

Note: *R*^2^ = 0.2457 for models with BMI and *R*^2^ = 0.2349 for models with FM%. All models were adjusted for age, education level, self-rated health and smoking.

**Table 4:** Estimated BMI and FM% when reallocating between 0 and 30 min from SB to MVPA.

Shift from SB to MVPA (min)	Predicted BMI (kg/m^2^)	Predicted FM (%)
0	28.15	38.46
5	27.83	38.00
10	27.54	37.60
15	27.29	37.23
20	27.05	36.89
25	26.84	36.57
30	26.64	36.28

Note: The mean composition was closed to 16 h.

**Table 5:** Estimated BMI and FM% when reallocating between 0 and 30 min from SB to LIPA.

Shift from SB to LIPA (min)	Predicted BMI (kg/m^2^)	Predicted FM (%)
0	28.15	38.46
5	28.13	38.40
10	28.10	38.36
15	28.08	38.29
20	28.06	38.23
25	28.04	38.17
30	28.01	38.11

Note: The mean composition was closed to 16 h.
